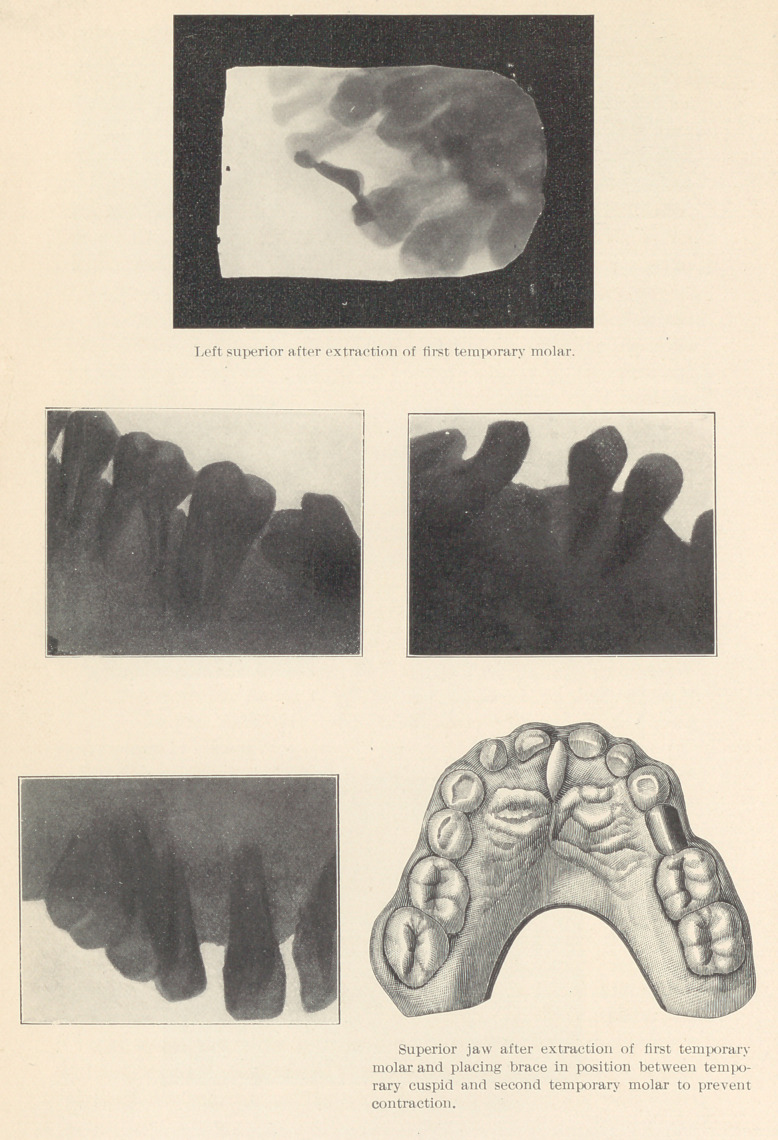# Persistently Retarded Temporary Teeth

**Published:** 1900-08

**Authors:** George S. Allan

**Affiliations:** New York


### International Dental Journal.

Vol. XXI. August, 1900. No. 8.

### Original Communications.[1]

### PERSISTENTLY RETARDED TEMPORARY TEETH.[2]

BY DR. GEORGE S. ALLAN, NEW YORK.

Mr. President and Gentlemen of The New York Institute of Stomatology,—My purpose in appearing before you this evening is not to read a paper, but simply to present a case: to give you such facts in regard to it as have come under my notice, something of its history, with what little I have been able to do to understand it, and to confess my general ignorance of the causes that brought about so abnormal a condition, and the many doubts and fears that have beset my path as to giving advice or attempting treatment.

I am not one of those who believe, with Pope, that "A little knowledge is a dangerous thing." On the contrary, I fully believe that a little knowledge is a mighty good thing to have handy for use; and I confess I am hungry for knowledge on this particular case, hence my bringing it to your notice.

I very much hope that, from the discussion that will take place when I have finished, or from the publishing of our proceedings, some valuable aid may be derived that will throw light on the

[1] The editor and publishers are not responsible for the views of authors of papers published in this department, nor for any claim to novelty, or otherwise, that may be made by them. No papers will be received for this department that have appeared in any other journal published in the country.

[2] Read before The New York Institute of Stomatology, March 6, 1900.

subject and help me to understand it. If good results do not follow, then it will go to show that the case and all things appertaining to it are as rare and curious as they appear to me.

On April 1, 1897, Master A. B., eleven years old, was brought to my office by his father for advice and treatment. Let me say just here that the lad is in every way, mentally and physically, except in that which I will presently refer to, strong and healthy. He is well developed for his years, and his father tells me he stands well in his school. He loves fun and sport, and, for a boy, takes kindly to his work.

On examination I found that I had to deal with a case of almost complete retention of the first teeth, upper and lower. In the upper jaw all the temporary teeth were in their proper places, firm and strong in their sockets, the two central incisors only yielding slightly to pressure. Between the centrals, however, there was a supernumerary, and this extra was a little more shaky than its neighbors. Back of the temporary second molars there was enough swelling and enlargement of the gums to show that the sixth-year molars were pressing forward and might reasonably be expected to erupt at an early date. On the palatine border of the alveolar ridge of the upper jaw on both sides the swelling was very pronounced, so much so that the thickness of the ridge was apparently nearly double what it should be. The width of the jaw was slightly below the normal. The depth was, however, about correct. The probe indicated a thickness of about three-eighths of an inch of gum tissues over the sixth-year molars, but this could not be definitely determined.

The lower jaw presented a somewhat different state of affairs. The temporary incisors had been cast off, and the permanent centrals were about a quarter of an inch through the gum. The thickness of the alveolar ridge was not so marked as in the upper jaw, in fact, not enough to attract attention, and the left sixth-year molar was in place, but not fully erupted. The occlusion of both jaws was perfect. All the temporary teeth were healthy and strong, of a dense yellow color, and hardly at all impaired by decay. The erupting sixth-year molar was not so fortunate, and showed signs of imperfect calcification and breaking down of its grinding surface.

The speech of the boy was not much affected by the small palate and lack of tongue-room. Still it was noticeable. So far as I can learn, the boy's first set of teeth indicated no departure from normal

November 18, 1899.

February 10, 1900.

Appliance used to draw out bicuspid tooth.

Left inferior before extraction of first temporary molar.

Left superior after extraction of first temporary molar.

Superior jaw after extraction of first temporary molar and placing brace in position between temporary cuspid and second temporary molar to prevent contraction.

conditions. They came about the right time,—a little tardy, not much,—and the two centrals were cast off and the two permanent centrals took their place in the regular course of time, so much so that this period of dentition attracted no attention. In order to account for so wide and exceptional a departure from natural conditions, the hereditary possibilities were first inquired into. But little light or information of value was obtained. Nothing similar or of like nature, more or less removed, was found to exist in any member of his family. His mother has a supernumerary back tooth between the left superior second bicuspid and first molar, but no other deviations from natural conditions. The father, however, has a most pronounced protrusion of the upper front teeth, accompanied with occlusion of the lower front teeth with the soft tissues covering the alveolar ridge just inside the upper centrals, and this peculiarity he has transmitted, most unfortunately, to two of his children. One or more of his sisters, I hear, had like peculiarities, which were remedied in early youth, but in no direction can there be found any history of tardy eruption of either sets of teeth or undue retention of the first set.

Seeking further light in a different channel, one nearer at hand, certain prenatal conditions of the mother not usual were mentioned by the parents, and should be alluded to here as having a possible bearing on the solution of the problem. The more so as they were followed by other abnormal conditions in the child. I cannot say that any great reliance can be placed on them, still they are of interest, and some of you may think they have value.

As stated by the mother they are as follows: "When three months pregnant, the physician in charge suspected I had developed diabetes, and he insisted on my giving up all sugar, all starchy fruits and food, excepting bread only. I lived almost entirely on meat, eggs, and green vegetables; no dessert of any kind; only oranges were allowed, as they contained so little sugar. This diet was faithfully kept up until the child was born. He was a delicate baby, with an abnormal opening in his head, extending from the middle of the forehead to the back of the head. This closed very slowly year by year until the child was four years old, when it became entirely closed. The child cut his first teeth when he was seven months old; the other teeth came slowly; the last baby tooth was cut when he was five years old. He was born May 13, 1886, so he is now nearly fourteen years old and is cutting his sixth-year molars." The above facts and data were given to me March 1, 1900.

No literature covering just such a case has come to my notice, so I make my report rather full in the hope that some one or more following me may find that it contains useful material, and that points of similarity may throw light on a difficult diagnosis.

I mentioned that the patient came to me first on April 1, 1897. Through some misunderstanding which I cannot explain I did not see him again till February 2, 1899. The parents thought I did not care to see him, for the reason that operative interference of any kind was impossible and a waiting policy only was proper. This was, in the main, correct, but not to the extent they thought. Nothing was done at first but make X-ray pictures. They were not very satisfactory, though they did show permanent teeth embedded in the maxillary bones and the roots of the temporary teeth of full length, with their fair proportions not in any way curtailed by the process of absorption. On the patient's second appearance it was evident that, left alone, no change for the better could be expected for many years, if ever. Nature unassisted either could not or would not give a helping hand. The two years of non-intervention left things practically as they were when first seen. Two more of the sixth-year molars had made their appearance, and the two lower centrals had increased their length to nearly full proportions.

The process of root-absorption was evident only by its complete absence, and the contemporary process of tooth eruption, while present, was painfully slow and tardy in commencing. All that could be done was a new effort to catch on to the case. New casts and X-ray pictures were made, and more consultations sought for. The first were helpful, but by no means decisive, and the latter only led to doubts and discord. One friend advised extraction and widening of the jaws; another said, "Let it alone; don't get into trouble." A third went over the whole ground, giving pros and cons, and came to the conclusion that he had no advice to give. Hardly any two gave the same advice or explanation. They agreed only in saying that they had never seen the like before or had they known of one having been reported. So, on general laws and principles, reliance only was to be placed with the added satisfaction of knowing that, whatever advice or course of treatment might be adopted, one would have to take great risks, and quite likely live to see the day when he would be sorry he had not advised and done otherwise.

After much study and thought and the placing before the parents of the possibilities of doing and not doing anything, I decided to have two teeth extracted and watch the result. The two left temporary molars were extracted on June 10, 1899, a few weeks before the boy, with his parents, sailed for Europe, to be away for four or five months. These teeth were extracted on account of their location being in the middle of the arch, and the probability that the first permanent bicuspids would naturally be most advanced. Immediately after extracting, two small braces were made and cemented to the teeth on the opposite sides of the vacant spaces to prevent any contraction, which, if it took place, as was probable if left alone, would certainly complicate matters if not make them decidedly worse.

My opinion was that if the permanent teeth were given a chance they might and probably would slowly erupt, and that the probabilities were strong enough to make it prudent to extract two temporary teeth in order to test the wisdom of my theory. The malformation and possibilities resulting therefrom were too positive not to make some effort to remedy it.

On December 14, 1899, seven months after the extraction of the two temporary teeth, other X-ray pictures were taken and careful examination and comparisons made with those taken at earlier dates. The net results, while not wholly satisfactory, indicate that the theory adopted and acted on was fairly well founded. A slow movement forward of the permanent teeth was indicated; a rapid one was not looked for. What may happen in the future remains to be seen. Comparison was made at this time of the width of the arch with the earlier casts. This measurement showed that the width of the arch was increased considerably,—fully an eighth of an inch.

The diagnosis of this case appears fairly simple and easy, the etiology and treatment obscure and doubtful; but how about the prognosis? Let the treatment or non-treatment, interference or non-interference, be what it will, what data have we with which to work or to predicate results?

To me this question seems more than a Chinese puzzle, and the more I turn it over in my mind the more hopeless and far-away does the solution appear. One does not like to work wholly in the dark or too far on experimental lines. I said I had no record of a like case. I made a mistake, as the letter I will now read shows. In essentials the two cases are closely related, but the pathological conditions of Dr. Howell's are wholly foreign to mine.

The heroic treatment adopted and carried out by Dr. Howell was, however, rewarded with the most fortunate results, which fully endorse the views that controlled my actions in the handling of my own.

## Figures and Tables

**Figure f1:**
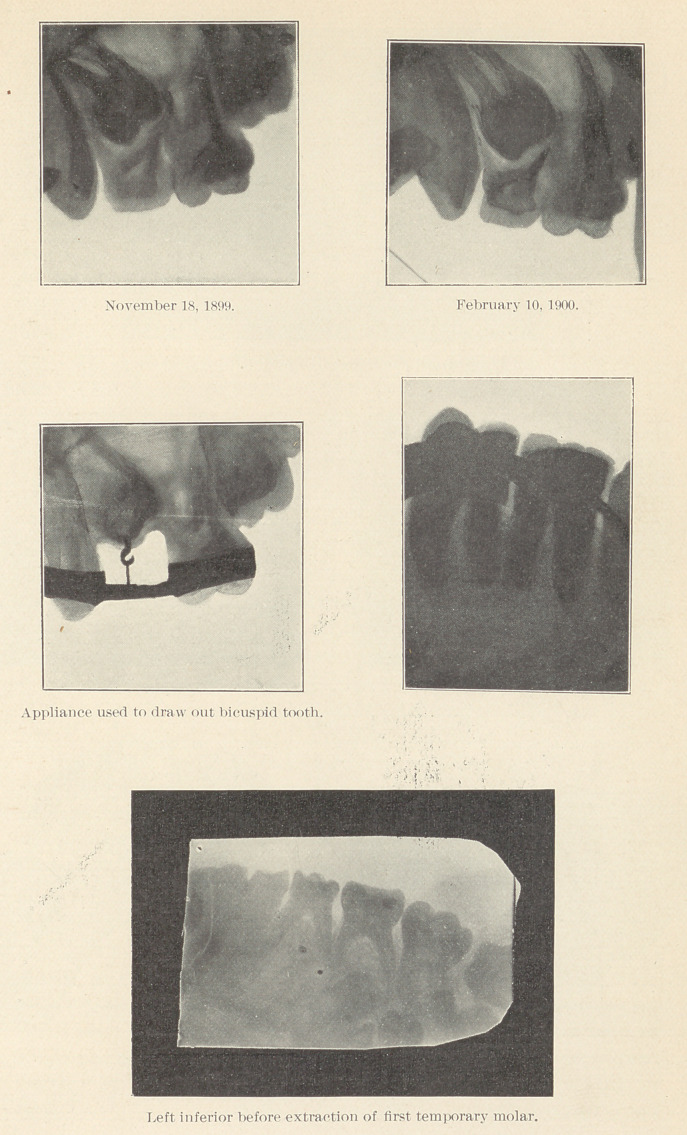


**Figure f2:**