# Correction

**Published:** 1900-12

**Authors:** 


### CORRECTION.

THROUGH a misunderstanding, illustrations 1, 2, and 3, accompanying Dr. Allan's paper in the August number, should have been placed with Dr. Weston A Price's communication in the report, in the same number, of the proceedings of The New York Institute of Stomatology, where they properly belonged.

